# The role of visual stimuli on standing posture in children with bilateral cerebral palsy

**DOI:** 10.1186/s12883-016-0676-2

**Published:** 2016-08-24

**Authors:** Cecilia Lidbeck, Åsa Bartonek, Priti Yadav, Kristina Tedroff, Per Åstrand, Kerstin Hellgren, Elena M. Gutierrez-Farewik

**Affiliations:** 1 Department of Women’s and Children’s Health, Karolinska Institutet, SE-171 76 Stockholm, Sweden; 2 Royal Institute of Technology, KTH Mechanics, Stockholm, Sweden; 3 Royal Institute of Technology, KTH BioMEx Center, Stockholm, Sweden; 4 Department of Clinical Neuroscience, Karolinska Institutet, SE-171 76 Stockholm, Sweden

**Keywords:** Cerebral palsy, Muscle activity, Postural orientation, Sensory disturbances, Standing ability, Visual function

## Abstract

**Background:**

In children with bilateral cerebral palsy (CP) maintaining a standing position can be difficult. The fundamental motor task of standing independently is achieved by an interaction between the visual, somatosensory, and vestibular systems. In CP, the motor disorders are commonly accompanied by sensory and perceptual disturbances. Our aims were to examine the influence of visual stimuli on standing posture in relation to standing ability.

**Methods:**

Three dimensional motion analysis with surface electromyography was recorded to describe body position, body movement, and muscle activity during three standing tasks: in a self-selected position, while blindfolded, and during an attention-demanding task. Participants were twenty-seven typically-developing (TD) children and 36 children with bilateral CP, of which 17 required support for standing (CP-SwS) and 19 stood without support (CP-SwoS).

**Results:**

All children with CP stood with a more flexed body position than the TD children, even more pronounced in the children in CP-SwS. While blindfolded, the CP-SwS group further flexed their hips and knees, and increased muscle activity in knee extensors. In contrast, the children in CP-SwoS maintained the same body position but increased calf muscle activity. During the attention-demanding task, the children in CP-SwoS stood with more still head and knee positions and with less muscle activity.

**Conclusions:**

Visual input was important for children with CP to maintain a standing position. Without visual input the children who required support dropped into a further crouched position. The somatosensory and vestibular systems alone could not provide enough information about the body position in space without visual cues as a reference frame. In the children who stood without support, an intensified visual stimulus enhanced the ability to maintain a quiet standing position. It may be that impairments in the sensory systems are major contributors to the difficulties to stand erect in children with CP.

## Background

Maintaining a stable standing position without support can be difficult in children with cerebral palsy (CP). Caused by an injury to the immature brain, CP is the most common impairment affecting motor development. Besides disorders of movement and posture, the accompanying disturbances of sensation and perception that commonly occur may interfere with the ability to function in daily life [1]. Almost one third of those affected have involvement of the lower limbs and a diagnosis of bilateral spastic CP [2]. The children’s self-initiated mobility, with emphasize on walking and sitting, is usually classified with the five-level gross motor function classification system (GMFCS) [3]. In terms of standing ability, most children who can stand without support are functioning in GMFCS level I or II, while children who require support to stand are in GMFCS level III or IV [4]. Children with bilateral CP tend to stand in a crouched position with a considerable amount of movements between body segments. This is even more pronounced in those who require support for standing [4]. In CP, standing posture has most commonly been studied in terms of postural stability in children who stood without support. In children with mild CP, postural deficits such as decreased stability and atypical postural sway are common [5, 6]. Difficulties achieving head stability and extensive head movements even while sitting have also been reported [7], as well as difficulties modulating lower limb muscle activity in task-specific and stability-provoking conditions during standing [8, 9].

The deviant gross motor function in children with CP is traditionally attributed to motor disorders alone, but this may not be the case. For example, one study reports that spasticity was found only minimally relates to gross motor function in children with bilateral CP [10]. Likewise, studies evaluating gross motor function before and after muscle tone reduction have shown no or limited functional gains despite spasticity reduction [11, 12]. In a previous study, we found that neither spasticity nor contractures could determine the need for support to stand in children with bilateral CP [4]. In another study, we have shown that muscle strength does not determine standing ability; strength in the lower limbs did not differ between children with CP who could or could not stand unsupported [13]. The development of standing ability may in fact be due to more complex combinations of both motor and sensory disorders.

The fundamental motor task of standing without support is achieved by a fusion of sensory information from the visual, somatosensory and vestibular systems. This multifaceted interaction enables perception of body position in space and is crucial for the control of body orientation and stability in standing. Maintaining a standing position and opposing gravity require an active alignment of body segments in relation to the environment and the task [14, 15].

Somatosensory disorders with proprioceptive deficits and thereby reduced ability to perceive limb positions have been associated with instability in standing in children with CP [16, 17]. Perception, defined as the ability to incorporate and interpret sensory information, may also be disturbed [1]. Perceptual impairments, such as easily induced startle reactions, freezing of posture, and averted eye gaze, have been found to strongly influence motor control strategies [18]. Impaired visual function is another sensory disturbance that frequently occurs. One study reported that more than 75 % of children with bilateral CP have neuro-ophthalmological disorders such as reduced visual acuity and oculomotor dysfunction [19]. To investigate the role of vision for the stability in standing, children are commonly tested with occluded vision. Typically-developing (TD) children and children with mild CP appear to be equally dependent on the visual system for stability; they increased sway while blindfolded to an equal extent [5, 6, 17]. It is worth noting that in these studies, some of the children with CP reacted to the blindfold with more apparent instability, which has been interpreted as an indication of sensory disturbances. Conversely, enhanced visual attention in the form of concurrent visual feedback has been reported to improve stability in standing in children with mild CP [6].

Since the variation in standing ability in children with CP could not be attributed to motor disorders alone, we were interested to find out whether the postural instability was associated with disturbances in the sensory system. The objective of this study was therefore to explore the influence of visual stimuli on standing posture. Our hypothesis was that difficulties in standing are related to need for visual stimuli; children with higher difficulties will be more dependent on visual input.

## Methods

### Participants

This study, investigating the influence of vision on standing posture in children with CP, was conducted at the Karolinska University Hospital, Stockholm, Sweden. The inclusion criteria were children and adolescents with bilateral CP between 6 and 17 years of age, GMFCS levels I-IV, ability to maintain standing with or without support for at least of 30 s, and ability to follow verbal instructions. Exclusion criteria were presence of dystonia, botulinum toxin injections or soft tissue surgery within the past six months, or skeletal surgery in the past year. Children were consecutively recruited through the neuropediatric department. A convenient sample of TD children constituted the control group. A total of 63 children and adolescents participated: 27 TD children – 11 girls and 16 boys, mean (SD) age 10.9 (3.5) years – and 36 children with bilateral CP – 16 females and 20 males, age 11.5 (3.0) years. The children with CP were divided into two groups based on need for support while standing: 17 stood with support (CP-SwS), and 19 stood without support (CP-SwoS). No differences were found between the TD children and the children with CP in age, gender, height, or weight (Table 1).

**Table 1:** Characteristics of children with bilateral CP standing with (CP-SwS) or without (CP-SwoS) support. No significant differences were found between groups in any parameter

	CP-SwoS (*n* = 19)	CP-SwS (*n* = 17)
Age, mean (SD) years	11.8 (2.7)	11.3 (3.4)
Female/male	8/11	8/9
Height, mean (SD), cm	147.8 (13.7)	139.5 (18.0)
Weight, mean (SD), kg	44.5 (15.5)	36.9 (13.3)
Gestational age, wk		
≤ 29	4	2
30–36	7	9
≥ 37	6	4
Unknown	2	2
GMFCS level:n	I:5, II:12, III:2	II:1, III:13, IV:3
Nero-ophthalmological abnormalities^a^		
Visual acuity < 0.33	1/17	1/16
Reduced visual field (hard to assess)	4 (1)/16	1 (7)/16
Dysmetric saccadic movements	11/16	11/16
Reduced stereopsis	8/16	11/16
Altered smooth pursuit movements	9/16	10/16
Strabismus	7/16	11/16
Instable fixation	3/15	5/15

^a^in number of children evaluated with each test

### Visual function

The children with CP underwent a neuro-ophthalmological examination on a separate occasion. Binocular visual acuity with visual charts at 3 m was categorized as either normal/near to normal (≥0.33 in decimal value) or moderate/severe/blind (<0.33 in decimal value) [20, 21]. Visual field was evaluated with the Stycar ball test, in which the patient reported when a white 3 cm diameter ball first became visible as it was moved inward from beyond the boundary of each quadrant of the visual field, or with the kinetic manual Goldmann Perimetry, documented as normal, hard to assess or reduced [22, 23]. The evaluation instruments were chosen based on each child’s ability to participate. Furthermore, oculomotor function was assessed by evaluating saccadic movements as normal or dysmetric, smooth pursuit movements as normal or altered, and to detect strabismus as present or absent. Fixation was qualitatively assessed as good or instable.

### Motion analysis

Standing posture was recorded with a three-dimensional eight-camera motion analysis system (Vicon MX40®, Oxford, UK) using a full-body biomechanical model and marker set (Plug-In-Gait, Vicon®) with reflective markers during standing on two force plates (Kistler®, Winterthur, Switzerland) embedded in the floor. Data from the more weight-bearing limb, as determined from force plate data during each child’s self-selected standing, was used for analysis. Body position angles were described by sagittal plane angles of the head, trunk and pelvis segments and the hip, knee, and ankle joints during the recorded time. Body movement ranges were described as ranges of joint movements, defined as the differences between maximum and minimum angles during the recorded time.

### EMG

Surface electromyography (sEMG) data was collected using a wireless EMG system (Noraxon®, USA) at a sample rate of 1000 Hz. Surface electrodes (Ambu®, Denmark) were placed bilaterally on the rectus femoris (RF), tibialis anterior (TA), medial gastrocnemius (MG) and soleus according to the surface SENIAM recommendations [24]. The raw sEMG signal was offset to zero and high-pass Butterworth filtered at 10 Hz. Root mean square (RMS) was determined over a 50-ms window, and the maximum RMS value was used for further analysis. Each muscle’s maximum RMS was then normalized to its corresponding value during the no-task standing condition and expressed as a percent.

### Procedure

The children stood barefoot on the force plates during the testing sessions. Those who required support held with a slightly flexed elbow position to a horizontal bar. Standing posture was recorded for 30 s during three standing tasks: a) no-task: in a self-selected position, b) blindfolded, and c) attention-task: while watching a video. The video was a film sequence of a child playing with a dog, shown on a 52 × 30 cm computer screen placed 1 m in front of the child. The purpose with the video was to provide a context external to the body which required focus of attention. Short sitting breaks between the testing conditions were taken when requested.

### Statistical analysis

Statistical analyses were carried out using commercially available software (SPSS v.21 Chicago, IL, USA). Data were normally distributed and were therefore analyzed with parametric statistics. One-way ANOVA tests with Bonferroni post hoc compensation for multiple comparisons were used to analyze body position and body movement ranges during the no-task standing condition among the three groups (TD, CP-SwoS, and CP-SwS). Paired *t*-tests were subsequently used to evaluate influence of vision (blindfolded-task and attention-task) on body position, body movements, and muscle activity within groups with the no-task condition as each child’s own reference. Chi-Square tests were used to compare whether incidence of ophthalmological findings varied between CP SwoS and CP SwS. Significance level was determined at the *p* < 0.05 level.

## Results

### Visual function

Ophthalmological examinations were performed in 32/36 children with CP: 16/19 in CP-SwoS and 16/17 in CP-SwS. Of the four children not assessed, one child (CP-SwoS) had an additional neurological disorder that excluded a valid assessment, two children (CP-SwoS) declined to attend the examination as it involved a further hospital visit, and one child (CP-SwS) had moved away of the area. Neuro-ophthalmological impairments were found in all children but four (one in CP-SwoS and three in CP-SwS). The children’s visual acuity was considered sufficient to see the film during attention-task in all children but one (CP-SwS). Ophthalmological status did not differ between CP-SwS and CP-SwoS (Table 1).

### Standing during the no-task condition

**Fig. 1:**
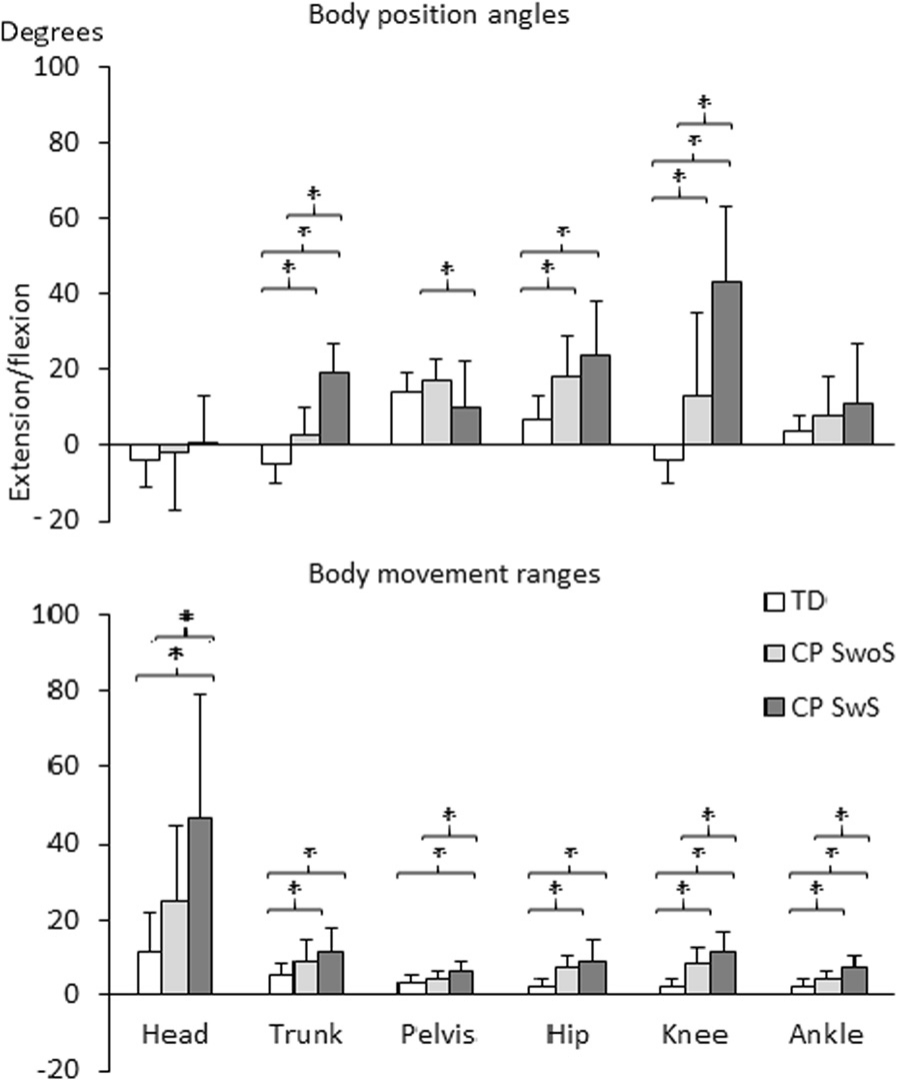
Illustration of sagittal plane body position angles and body movement ranges (ranges of joint movements between the maximum and minimum angles) during the no-task standing condition in the TD children and the children with CP standing with (CP SwS) or without support (CP SwoS). Negative values (−) indicate extended position of the head, posteriorly tilted trunk, and knee hyperextension. An asterisk (*) indicates significant differences between the groups (*p* < 0.05)

Standing posture during the no-task condition was examined in all 63 children (Table 2 and Fig. 1). All three groups of children stood with a similar upright position of the head (*p* = 0.390) and with a similar few degrees of dorsiflexion in the ankle (*p* = 0.084). There were differences among groups in the position of the trunk (*p* < 0.001), pelvis (*p* = 0.032), hip (*p* < 0.001), and knee (*p* < 0.001). Compared to the TD children, the children in CP-SwoS and CP-SwS stood with anteriorly tilted trunks (*p* < 0.001 for both), with more flexed hips (*p* = 0.001 and *p* < 0.001, respectively), and with more flexed knees (*p* = 0.003 and *p* < 0.001, respectively). Compared to CP-SwoS, the children in CP-SwS stood with more anteriorly tilted trunk (*p* < 0.001), with less anteriorly pelvis tilt (*p* = 0.029) and more flexed knees (*p* < 0.001).

**Table 2:** Standing posture (body position, body movements and muscle activity) in typically developing (TD) children and in children with bilateral CP standing with (SwS) or without (SwoS) support, during three standing tasks: no-task (NT), blindfolded-task (BT), and attention-task (AT). EMG muscle activity in the rectus femoris (RF), tibialis anterior (TA), medial gastrocnemius (MG), and soleus normalized to the NT condition. Paired *t*-tests were used to examine the influence of vision (BT and AT) with respect to the NT condition. Significant differences are bolded (*p* < 0.05)

	TD					SwoS					SwS				
	NT (*n* = 27)	BT (*n* = 27)	AT (*n* = 27)	NT-BT *p*	NT-AT *p*	NT (*n* = 19)	BT (*n* = 18)	AT (*n* = 19)	NT-BT *p*	NT-AT *p*	NT (*n* = 17)	BT (*n* = 17)	AT (*n* = 16)	NT-BT *p*	NT-AT *p*
Body position angles degrees, mean (SD)															
Head	−4^a^ (7)	−7^a^ (5)	−6^a^ (5)	**0.028**	0.114	−2^a^ (15)	−2^a^ (12)	−5^a^ (9)	0.619	0.216	1 (12)	−5^a^ (13)	−6^a^ (9)	0.119	**0.015**
Trunk	−5^b^ (5)	−5^b^ (5)	−5^b^ (5)	0.787	0.831	3 (7)	3 (7)	2 (8)	0.463	0.098	19 (8)	20 (10)	19 (9)	0.537	0.858
Pelvis	14 (5)	14 (5)	14 (5)	0.644	0.552	17 (6)	17 (6)	15 (7)	0.085	0.327	10 (12)	9 (12)	9 (14)	0.777	0.590
Hip	7 (6)	6 (6)	6 (6)	0.161	0.070	18 (11)	18 (12)	17 (11)	0.734	0.277	24 (14)	28 (15)	25 (15)	**0.014**	0.906
Knee	−4^c^ (6)	−5^c^ (6)	−6^c^ (7)	0.505	0.142	13 (22)	12 (23)	12 (23)	0.646	0.576	43 (20)	47 (22)	45 (21)	**0.016**	0.928
Ankle	4 (4)	4 (4)	3 (4)	0.924	0.334	8 (10)	8 (11)	8 (11)	0.285	0.837	11 (16)	10 (20)	10 (17)	0.928	0.843
Body movements ranges degrees, mean (SD)															
Head	12 (10)	8 (6)	8 (5)	0.056	0.053	25 (20)	16 (12)	16 (9)	**0.037**	**0.038**	47 (32)	35 (26)	29 (25)	0.106	**0.012**
Trunk	5 (3)	4 (2)	4 (3)	0.064	0.160	9 (6)	6 (2)	7 (4)	0.064	0.281	12 (6)	14 (10)	14 (7)	0.377	0.215
Pelvis	3 (2)	2 (1)	2 (2)	0.071	0.457	4 (2)	4 (1)	4 (2)	0.245	0.753	6 (3)	6 (3)	7 (3)	0.731	0.234
Hip	2 (2)	2 (1)	3 (3)	0.173	0.284	7 (4)	6 (4)	6 (4)	0.210	0.181	9 (6)	10 (6)	9 (5)	0.761	0.817
Knee	2 (2)	2 (1)	2 (2)	0.824	0.823	8 (5)	7 (6)	6 (3)	0.496	**0.021**	12 (5)	11 (5)	11 (5)	0.263	0.419
Ankle	2 (2)	2 (1)	2 (1)	0.739	0.956	4 (2)	4 (3)	4 (2)	0.771	0.429	7 (4)	7 (5)	6 (5)	0.807	0.702
Muscle activity % of NT															
RF		−2 (21)	−1 (19)	0.694	0.875		0 (23)	−8 (11)	0.968	**0.007**		12 (20)	−2 (16)	**0.029**	0.689
TA		−3 (26)	2 (35)	0.530	0.774		17 (43)	−8 (24)	0.109	0.172		−1 (15)	−5 (11)	0.813	0.068
MG		37 (65)	5 (29)	**0.006**	0.328		34 (61)	0 (35)	**0.030**	0.964		−4 (17)	−6 (14)	0.322	0.114
Soleus		16 (32)	−1 (22)	**0.014**	0.879		13 (34)	−11 (19)	0.123	**0.018**		−6 (13)	−5 (10)	0.069	0.054

^a^Head extension, ^b^Posteriorly tilted trunk, ^c^Knee hyperextension

Among the three groups, there were differences in body movement ranges in all segments and joints: the head (*p* < 0.001), trunk (*p* < 0.001), pelvis (*p* < 0.001), hip (*p* < 0.001), knee (*p* < 0.001), and ankle (*p* < 0.001). The TD and CP-SwoS children stood with similarly small movements of the head (*p* = 0.120) and pelvis (*p* = 0.157), whereas CP-SwoS stood with greater movements of the trunk (*p* = 0.020), hip (*p* = 0.002), knee (*p* < 0.001) and ankle (*p* = 0.003). Compared to the TD children, the children in CP-SwS stood with greater movements in all body segments and joints: the head (*p* < 0.001), trunk (*p* < 0.001), pelvis (*p* < 0.001), hip (*p* < 0.001), the knee (*p* < 0.001), and ankle (*p* < 0.001). The children in CP-SwoS and CP-SwS stood with similar movements of the trunk (*p* = 0.077) and hip (*p* = 0.268), whereas children in CP-SwS stood with more movements of the head (*p* = 0.007), pelvis (*p* = 0.023), knee (*p* = 0.009), and ankle (*p* = 0.001) (Fig. 1).

### Blindfolded-task vs no-task conditions

**Fig. 2:**
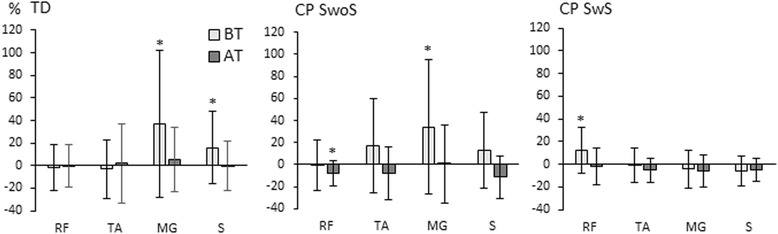
Illustration of EMG muscle activity (%) normalized to the no-task (NT) standing condition in the rectus femoris (RF), tibialis anterior (TA), medial gastrocnemius (MG), and soleus, during the blindfolded-task (BT) and attention-task (AT). An asterisk (*) indicates significant differences between the NT and the two visual conditions BT and AT respectively (*p* < 0.05)

All children stood blindfolded but one child (CP-SwoS) who declined (Table 2 and Fig. 2). While blindfolded, the TD children stood with the head in a more upright position (*p* = 0.028) than in the no-task condition. Body movement ranges were unchanged, but muscle activity in the lower limbs increased in the MG (*p* = 0.006) and soleus (*p* = 0.014). The children in CP-SwoS stood with unchanged body position angles and less movement range of the head (*p* = 0.037), but greater muscle activity in the MG (*p* = 0.030). While blindfolded, the children in CP-SwS stood with more flexed hips (*p* = 0.014) and knees (*p* = 0.016). Body movement ranges were unchanged, but muscle activity increased in the RF (*p* = 0.029).

### Attention-task vs no-task conditions

All children were requested to watch the video film during the attention-task (Table 2 and Fig. 2). Data from the child (CP-SwS) with poor visual acuity was excluded. During the attention-task, the TD children stood with unchanged body position angles, body movement ranges, and muscle activity in the lower limbs. The children in CP-SwoS stood with unchanged body position angles, with less movement ranges of the head (*p* = 0.038) and knee (*p* = 0.021), and with less muscle activity in the RF (*p* = 0.007) and soleus (*p* = 0.036). During the attention-task, the children in CP-SwS stood with the head in a more upright (*p* = 0.015) and more still (*p* = 0.012) position. The muscle activity was unchanged.

## Discussion

In children with bilateral CP, visual input influenced standing posture differently depending on whether or not the children required support to stand. This indicates that impairments in the sensory systems contribute to the increased difficulties maintaining an erect standing position, and that the ability to stand without support may depend more on different sensory disturbances than on differences in motor disorders.

In this study, children with CP throughout the spectrum of GMFCS levels I-IV were included, provided they could stand barefoot with or without support. As previously reported, the children with CP stood with a flexed body position, which was more apparent in the children who required support for standing [4]. When blindfolded, all the children who stood without support, whether TD or with CP, maintained their habitual body position, but their calf muscle activity increased. In contrast, the children in CP-SwS (requiring support for standing) stood with an even more flexed body position with increased muscle activity in the knee extensors but not around the ankle.

Vision provides a reference frame external to the body, based on cues in the near environment. These cues may help compensate for insufficient function in the somatosensory or vestibular systems [25]. In the present study, standing while blindfolded was demanding for all children, even those who could stand unsupported. Compared to the no-task condition their calf muscle activity increased by an average of more than 30 %. Thereby, children in both the TD and the CP-SwoS groups were able to adapt their posture to the new environmental demands by increasing muscle activity around the ankle only. The somatosensory and vestibular systems in the CP-SwoS group provided information about the body’s position in space that was apparently sufficient to maintain their habitual standing position without input from the visual system. In contrast, the children in the CP-SwS group used another strategy; when visual input was occluded, the already flexed body position became even more flexed and muscle activity increased in the knee extensors. Thus, without visual cues from the close environment, the children CP-SwS could not fully maintain their body position. Reduced ability to maintain posture while blindfolded indicates a loss of proprioception. In an earlier study, impaired joint position sense was linked to instability and increased sway during standing in some children with mild CP [17]. In the present study, impaired sense to detect the changes in body position might be associated with difficulties to adapt the posture with calf muscle activity in children in CP-SwS. The posture with increased hip and knee flexion in children in CP-SwS while blindfolded could, then, be considered as compensation due to somatosensory disturbances. However, information from both the somatosensory and vestibular systems contribute to more complex processes, such as perceiving the body position in space required for postural orientation in upright standing [25, 26].

Impairments in the perceptual system have been found to be associated with inefficient postural responses in children with bilateral CP in a study in which stability limits while sitting were provoked [27]. In another study, freezing of posture with a blocked rigid body position occurring during balance-provoking activities, was deemed attributable to a perceptual impairment in children with bilateral CP [18]. In our study, the blindfold can be considered provocative for children’s standing ability. Thus, the increased quadriceps muscle activity in the CP-SwS group could be an indication of co-contraction or co-activity elicited by perception of insecurity despite the handheld support. However, several studies have found increased levels of co-activity during provocative conditions in children with CP [28, 29]. In our study the increased muscle activity and flexed body position while blindfolded could be caused by sensory impairments. The increased muscle activity in children in CP-SwS may also be a consequence of the greater demands on muscle contraction or co-contraction often seen in CP [28]. Since children with CP in general are weaker than their typically-developing peers, one might hypothesize that the difficulty in adapting posture when environmental demands are increased is due to muscle weakness. However in a recent study we found that the ability to stand without support did not depend on muscle strength in children with bilateral CP, thus refuting that hypothesis [13].

The use of visual stimuli to improve control of movements and posture in children with mild CP has been suggested [6, 30]. In the present study, we investigated not only how an occluded, but even how an intensified visual stimulus influenced standing posture by asking the children to watch a short movie sequence. As could be expected in children with intact sensory motor systems, the TD children could watch the movie without any alterations in posture. Remarkably, posture in children with in CP-SwoS was aided by viewing the film; they stood more still, with less knee movement and with less activity in the knee extensors and calf muscles. The presence of the intensified visual attention cues seemed to improve posture, at least to some extent, in the children who could adapt to the new environment with muscle activity. In our study, impaired proprioception was most likely present even in the children with milder CP, as in the study by Wingert et al. [16].

Vision is not only important for standing posture, but also for the position of the head, particularly when sensory information is attenuated. In a study that investigated head stability during quiet sitting while vision was manipulated, it was found that children with CP have difficulties to stabilize the head, and that deficits worsened when the children were asked to close their eyes [7]. In accordance with these findings, we found a considerable amount of head movements in children with bilateral CP; in CP-SwS head tilt position varied almost 50° during the no-task condition. A notable finding was that children with CP maintained head position more easily while blindfolded, though significant only in the CP-SwoS group. In addition, all children with CP stood with less head movement while watching the video. Both tasks require increased focus – one visual and the other somatosensory – which may explain the more quiet head position.

Neuro-ophthalmological findings, pathological or aberrant, were confirmed in most of the children with CP, and this was equally distributed in both groups. Therefore, the difficulties that the CP-SwS group had to maintain a standing position could not be attributed to impaired vision. In general, posture has been more frequently studied in children with mild CP, typically GMFCS levels I-II, than in children with more extensive gross motor function difficulties. Analyzing posture in children who require external support can be difficult, and it was not possible to assess whether the amount of handheld support that was required varied during the different standing tasks, which can be considered a limitation.

## Conclusions

In this study, visual stimuli influenced standing posture in children with CP, wherein children who required support to stand deviated their posture more when the visual stimulus was removed than those who could stand without support. This indicates that their somatosensory and vestibular systems alone could not provide enough information about body position in space; they also required assistance from the visual system. An intensified visual stimulus enhanced the ability to maintain a quiet position in the children who could stand without support. These children’s ability to adapt their posture to different environmental demands with muscle activity, both with and without visual stimuli, enables them to stand without support. It may be that impairments in the sensory systems contribute to difficulties in standing erect in children with CP.

## Abbreviations

CP: Cerebral palsy
CP-SwoS: Children with CP standing without support
CP-SwS: Children with CP requiring support for standing
GMFCS: Gross motor function classification system
MG: Medial gastrocnemius muscle
RF: Rectus femoris muscle
RMS: Root mean square
sEMG: Surface electromyography
TA: Tibialis anterior muscle
TD: Typically-developing
